# The impact of biochar on wood-inhabiting bacterial community and its function in a boreal pine forest

**DOI:** 10.1186/s40793-022-00439-9

**Published:** 2022-08-30

**Authors:** Zhao-lei Qu, Xiao-li Li, Yan Ge, Marjo Palviainen, Xuan Zhou, Jussi Heinonsalo, Frank Berninger, Jukka Pumpanen, Kajar Köster, Hui Sun

**Affiliations:** 1 Collaborative Innovation Center of Sustainable Forestry in Southern China, College of Forestry, Nanjing Forestry University, Nanjing, 210037 China; 2 Department of Forest Sciences, University of Helsinki, Latokartanonkaari 7, P.O. Box 27, 00014 Helsinki, Finland; 3 Department of Environmental and Biological Sciences, University of Eastern Finland, Yliopistonranta 1 E, P. O. Box 1627, 70211 Kuopio, Finland

**Keywords:** Biochar application, Pyrolysis temperature, Wood degradation, Bacterial community and function, Boreal pine forests

## Abstract

Biochar is considered to be a possible means of carbon sequestration to alleviate climate change. However, the dynamics of the microbial community during wood decomposition after biochar application remain poorly understood. In this study, the wood-inhabiting bacterial community composition and its potential functions during a two-year decomposition period after the addition of different amounts of biochar (0.5 kg m^−2^ and 1.0 kg m^−2^), and at different biochar pyrolysis temperatures (500 °C and 650 °C), in a boreal Scots pine forest, were analyzed using Illumina NovaSeq sequencing combined with Functional Annotation of Prokaryotic Taxa (FAPROTAX). The results showed that the wood decomposition rates increased after biochar addition to the soil surface in the second year. Treatment with biochar produced at high temperatures increased the diversity of wood-inhabiting bacteria more than that produced at low temperatures (*P* < 0.05). The wood-inhabiting bacterial diversity and species richness decreased with decomposition time. The biochar treatments changed the wood-inhabiting bacterial community structure during the decomposition period. The pyrolysis temperature and the amount of applied biochar had no effect on the bacterial community structure but shifted the abundance of certain bacterial taxa. Similarly, biochar application shifted the wood-inhabiting bacterial community function in the first year, but not in the second year. The wood-inhabiting bacterial community and function were affected by soil pH, soil water content, and soil total nitrogen. The results provide useful information on biochar application for future forest management practices. Long-term monitoring is needed to better understand the effects of biochar application on nutrient cycling in boreal forests.

**Supplementary Information:**

The online version contains supplementary material available at 10.1186/s40793-022-00439-9.

## Introduction

Biochar is carbon-rich, highly aromatic, and stable solid material made of crop waste, wood, and other biological materials under anaerobic conditions and at high pyrolysis temperatures (usually < 700 °C) [1, 2] It is an effective material to boost soil fertility, and it may also be effective for carbon sequestration to mitigate climate change in forest ecological systems [3, 4]. Biochar application can increase carbon storage, the C/N ratio, and water content in boreal forest soils [5], and in general can increase the net carbon input of northern forests [6]. Soil nitrogen exists in organic form in boreal forests, and the low nitrogen mineralization rate limits tree growth [7]. Our previous study from the same study area showed that the application of biochar in boreal forests can also increase the net nitrogen mineralization and nitrification rates and increase tree growth [8].

Organic matter decomposition is one of the key processes of nutrient cycling in forest ecosystems [9]. The litter decomposition is directly linked to microbial activities, which alter the chemical compounds of litter and regulate the dynamics of soil carbon and nitrogen [10]. The microbial community in litter might be influenced not only by the litter type, but also by the properties of the surrounding soil, vegetation, and soil microorganisms [11]. Bacteria make up the majority of soil microorganisms, accounting for over 80%, and are vulnerable to variations in pH and the availability of C sources [12]. They are one of the earliest organisms to colonize dead wood and metabolize easily degradable and available substrates [13], which can affect the structure of wood during degradation [14]. In the initial stage of wood decay, bacteria likely undergo a succession before the fungal occupation of the microbial community [15]. It has been found that changes in the soil bacterial community composition can significantly impact the wood-associated bacterial structure [16]. A previous study showed that the bacterial community undergoes a series of colonizations in the course of the decomposition of wood, and Proteobacteria, Actinomycetes, and Bacteroides are the most abundant taxa [17].

Biochar application in a forest can both directly and indirectly affect the physical and chemical properties of forest soil, which changes the soil microbial abundance, composition, and function [18]. It has been shown that biochar application can increase soil microbial biomass and significantly shift soil microbial community composition due to the special properties of biochar [19]. The properties of biochar depend on the production method, pyrolysis temperature, and raw material type [20] It has been reported that biochar produced at relatively high temperatures (600–700 °C) has a high proportion of aromatic C, large porosity, and a low cation exchange capacity [21]. In addition, the amount of biochar applied also affects the growth of trees [22]. The application of 0.5–1 kg m^−2^ of woody biochar can significantly increase the growth of dominant tree species in the three years after biochar application [3].

In recent years, many studies have focused on the effects of biochar application on organic matter decomposition. For example, Wardle et al. observed, in a 10-year litter bag experiment, that biochar produced by wildfire led to the loss of forest humus [23]. The addition of biochar can promote litter decomposition in temperate forests by increasing the soil water content [24]. Abiven et al. found that biochar does not always stimulate the decomposition of litter with increasing application time. Instead, the decomposition may depend on other factors, such as soil properties and microorganisms [25]. Biochar application in northern forests may increase the decomposition of larch roots, and charcoal may promote the decomposition of organic matter in undergrowth plant residues [23]. However, the dynamics of the microbial community during wood decomposition after biochar application remain poorly understood. Such information could help to better understand the potential effects of biochar on the nutrient cycle, and provide information on the response of forests to biochar application. Therefore, in this study, we investigated the responses of a wood-inhabiting bacteria community and its functions during wood degradation. We used the litter mesh bag embedding method with different biochar treatments in a boreal pine forest. The hypothesis here is that the pyrolysis temperature and the application amount of biochar would affect the structure and function of wood-inhabiting bacteria during wood degradation. The aims of this study were to explore the effect of (1) biochar produced at different temperatures (500 °C and 650 °C); (2) the biochar application amount (0.5 kg m^−2^ and 1.0 kg m^−2^); and 3) the wood degradation time (one and two years after application) on the wood-inhabiting bacterial community composition and functions.

## Materials and methods

### Study sites and biochar treatment

The experimental site was at the Hyytiälä Forestry Field Station in southern Finland (61°48′N, 24°18′E, 181 m a.s.l.). The experimental plots were located around 20-year-old Scots pine (*Pinus sylvestris* L.) young forest land, which was naturally regenerated. A detailed description of the experimental setup can be found in Palviainen et al. [26]. The soil in the area is coarse sand and the forest site type is the low-fertility xeric type, and the terrain is flat. The ground vegetation is dominated by *Vaccinium vitis-idaea* L., *Callunavulgaris* (L.) Hull., *Empetrum nigrum* L., and *V. myrtillus* L.,mosses (*Pleurozium schreberi* (Brid.) Mitt., *Hylocomium splendens* (Hedw.) Schimp., and some lichens (*Cladina* sp.) [27]. The long-term annual average temperature in this area is 3.5 °C, with an average annual precipitation of 700 mm, and the length of snow cover is 145–160 days (1981–2010) [28].

The experimental random block design was adopted, with three replicates (referred to as blocks) and five plots (15 m × 15 m squares) within each block [29]. The blocks were separated by several hundred meters and belonged to different forests, to avoid pseudo replication. The distance between each plot was set to 10 m, and a 2.5-m-wide buffer around the edge of the plot was not used for measurements. The biochar was produced from Norway spruce (*Picea abies* (L.) H. Karst) wood chips at 500 °C and 650 °C (manufactured by Sonnenerde GmbH, Riedlingsdorf, Austria) using the Pyreg process (Pyreg GmbH, Doerth, Germany). The particle size of the biochar used in this experiment was 5 to 10 mm [29]. Two different biochar amounts were applied to each plot: 0.5 kg m^−2^ and 1.0 kg m^−2^. Therefore, there were five treatments in total in each block, namely 0.5 kg m^−2^ biochar produced at 500 °C (T1A1) and 1 kg.m^−2^ (T1A2), respectively; 0.5 kg m^−2^ (T2A1) biochar produced at 650 °C and 1 kg m^−2^ (T2A2), respectively; and the control treatment without biochar application (C). In May, 2015, biochar was spread on top of the vegetation on the surface of the soil at one time, in order to avoid disturbing the soil and damaging plant roots. The biochar application amounts were typical and economically feasible biochar application amounts in forests [30].

### Wood mesh bag embedding and sampling

Small dry wood cubes (1 × 1 × 1 cm) of Norway spruce (*Picea abies* (L.) H. Karst) were used for mesh bag embedding. The wood cubes were cut from a single plank from a dried spruce timber. Two wood cubes were put into a mesh bag, one of which was used for measuring the decomposition rate (mass loss), and the other for determining microbial community and functions. The wood cube for the decomposition study in each bag was marked by cutting one corner away, and its dry weight (g_0_; dried for 72 h at 65 °C) was taken before burying, and wood cubes used to measure microorganisms are subjected to the same drying process. Six mesh bags containing wood cubes were buried in the litter layer in each plot in June 2016, resulting in 30 mesh bags in each of the five treatments. Three mesh bags were randomly taken from each treatment in June 2017 and 2018. A total of 45 mesh bags were taken out and carried on ice to the laboratory each year. The samples were stored at − 20 °C until further processing.

The soil samples (0–10 cm) were collected close to the mesh bags containing wood blocks in 2017 and 2018 at the same time the mesh bags were collected. Three soil samples were collected from the organic layer from each plot using a stainless-steel soil corer (diameter 5.5 cm). The samples were stored at − 20 °C until further processing.

### Wood degradation rate and soil analyses

The pre-weighted wood cubes were placed in an oven at 65 °C for 72 h until a constant weight (g_1_) was reached, after the roots and soil materials on the surface of the wood cube were removed. The wood degradation rate refers to the dry weight loss of the wood cube after a period of decomposition (one year) between the initial and eventual dry weight of the cube (g_0_–g_1_). The fresh soil sample was dried at 105 °C for 24 h, and then the soil water content was measured. After the soil samples were air-dried and sieved through a 2 mm (or further through a 0.25 mm) sieve for determination of soil pH, soil organic matter, and soil total nitrogen.

### Sample DNA extraction, amplification of the 16S rRNA gene region, and Illumina NovaSeq sequencing

Each wood cube was ground in liquid nitrogen with a grinder (Kinematica, Switzerland) after removal of roots and soil materials on the surface, before after each sample is ground, clean the grinder by spraying and wiping with alcohol. 0.3 g (fresh weight) of each ground sample was used to extract the total genomic DNA using a DNA kit (TIANGEN BIOTECH (BEIJING) CO., LTD, Beijing, China), according to the DNA extraction instructions. An initial lysis process was carried out to the ground samples, including putting 700μL of GP1 (mercaptoethanol) preheated at 65 °C into the ground sample, followed by mixing the suspension evenly and then putting in a water bath at 65 °C for 20 min. The extracted DNA was quantitatively analyzed by NanoDrop-1000 spectrometer (NanoDrop Technologies, Wilmington, DE, USA) and the DNA quality with A260/A280 ration ranging from 1.8 to 2.0 was selected for subsequent analysis. PCR amplification of the V3–V4 region of the bacterial 16S rRNA gene was carried out with primers 338F (ACTCCTACGGGAGGCAGCAG) and 806R (GGACTACHVGGGTWTCTAAT) [31]. PCR was carried out in 50 μL reaction mixtures with the following components: 25 µL 2 × Premix Taq, 1 µL Forward Primer (10 µM), 1 µL Reverse Primer (10 µM), 50 ng Template DNA, and nuclease-free water added to constant volume. The PCR reaction parameters were as follows: 94 °C for 5 min, 30 cycles of 94 °C for 30 s, 52 °C for 30 s, 72 °C for 30 s, and 72 °C for 10 min. The PCR products were detected via 1% agarose gel electrophoresis. The PCR mixture was recovered using an E.Z.N.A. ® Gel Extraction Kit (Omega, USA) Gel recovery Kit, and the target DNA fragment was eluted with TE buffer. The DNA concentration was measured using a Nano-drop ND-1000 spectrophotometer, and PCR products were sequenced by the Illumina Nova 6000 platform at Majorbio (PE = 250) platform of Guangdong Magigene Biotechnology Co., Ltd. (Guangzhou, China). The raw sequences were uploaded in the National Center for Biotechnology Information (NCBI) with the accession number PRJNA797190.

### Sequence data processing and statistical analysis

The raw sequence data were processed according to the standard operating procedure (SOP) of Mothur software version 1.45.3 [32]. Under this SOP, after merging the reads R1 and R2, a sequence will be truncated if it contains: (i) ambiguous (N) bases; (ii) homopolymers longer than eight nucleotides; (iii) an average quality score lower than 25; (iv) chimeras (detected using Chimera uchim in Mothur); and (v) fewer than 200 nucleotides. The commands used to denoise and clean the sequences included fastp v.0.14.1 to remove the adapter and barcode sequences, trim.seqs to check the quality of sequencing errors, and pcr.seqs and chimera.uchime to check PCR errors and chimeras, respectively. Then, the sequences were pre-clustered with 6 bp differences by the Mothur pre-clustering method, and were clustered with 97% similarity to form operational taxonomic units (OTUs) [33, 34]. OTU with frequencies of less than 10 in all samples were deleted. The sequences were assigned to taxonomic groups with an 80% bootstrap confidence using the RDP Naïve Bayesian rRNA Classifier tool version 2.0. [35]. Sequences assigned to the plant chloroplast and non-bacteria domain were filtered out. Functional Annotation of Prokaryotic Taxa (FAPROTAX) was used to predict the biogeochemical cycle of the environmental samples, especially the cycle of carbon, nitrogen, phosphorus, with the default output function table, and we compared the dataset obtained by classification and the automatic function classifier FAPROTAX (script version 1.1 and database version 1.0) to determine the function group [36].

A total of 5,323,959 sequences were obtained after denoising and quality control, and the number of sequences of the samples ranged from 46,409 to 79,003, with an average of 59,155 ± 5011 (mean ± standard deviation). The minimum sample size of all samples (46,409) was used for random sampling and diversity indices calculation, including. Species richness estimation (Sobs, observed species), community diversity (Invsimpson), and community evenness (Simpson evenness) [37]. The centered log-ratio (CLR) transformation was used to convert value on raw abundance of functions data. One-way (ANOVA) and multivariate analysis of variance (MANOVA) were used to compare the differences in diversity and function structure of wood-inhabiting bacterial communities in different biochar treatments. The visualization and detection of differences in community structure and function were based on Canonical analysis of principal coordinates (CAP) based on Bray–Curtis dissimilarity at OTU level was used as ordination method and permutational analysis of variance (PERMANOVA) with Bray–Curtis dissimilarity after 9999 permutations. Venn diagrams (http://bioinformatics.psb.ugent.be/webtools/Venn/) were constructed using normalized data, which can intuitively show shared and unique OTUs among multiple samples. The relationship between the community structure or functional structure and the environmental factors was detected by distance-based linear model (DistLM) (selection procedure was exhaustive search using all variable combinations, and selection criteria was Akaike's Information Criterion (ACI), and the outcome p values in the supplementary table based on forcing inclusion of all specified variables). All the analyses were carried out by using PRIMER 7 [38]and SPSS.22.

## Results

### Wood decomposition rate in different biochar treatments

**Fig. 1:**
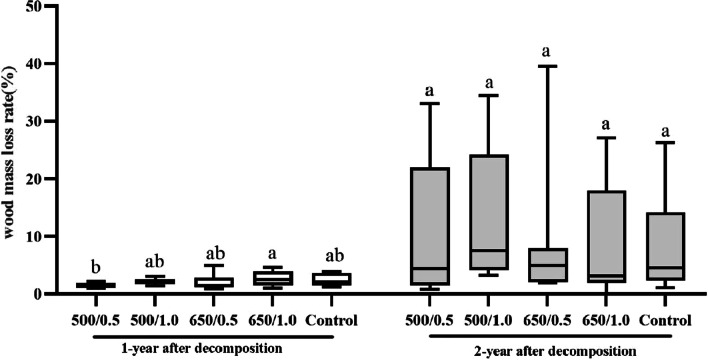
Wood loss after one and two years of decomposition in different biochar treatments. The number before and after "/" represents the pyrolysis temperature of the biochar (°C) and application amount of the biochar (kg m^−2^), Lowercase letters were used to indicate the significant difference (*P* < 0.05) among the five treatments within the same year after biochar application

The wood decomposition rate did not differ between the biochar treatments and the control in both the first and the second year (Fig. 1). The wood decomposition rates decreased with the increase in biochar pyrolysis temperature, and increased with biochar application amount (Fig. 1). In the five treatments, with the extension of wood degradation time, the degradation rate of wood increased significantly (*P* < 0.05) (Additional file 1: Table S2).

### Wood-inhabiting bacteria community diversity in different biochar treatments

**Fig. 2:**
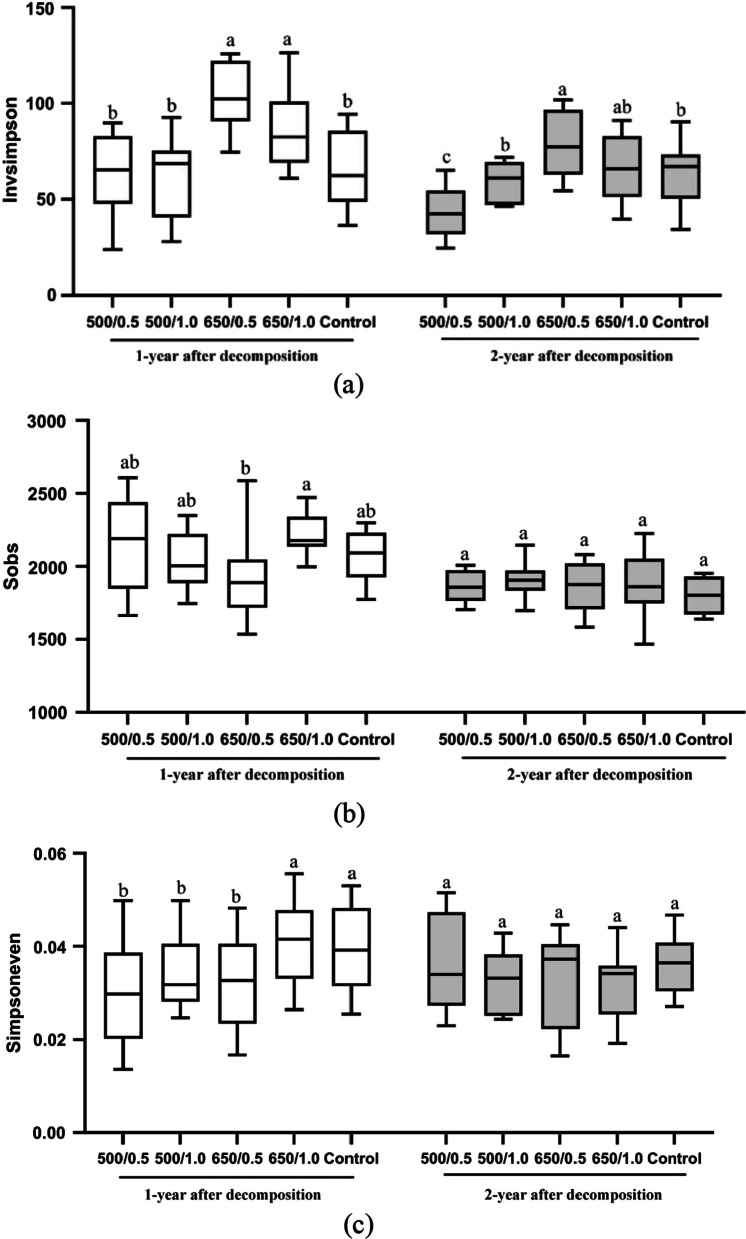
The wood-inhabiting bacterial community **a** diversity, **b** richness, and **c** evenness after one and two years of decomposition in different biochar treatments. The number before and after "/" represents the pyrolysis temperature of biochar (°C) and application amount of biochar (kg m^−2^), Lowercase letters were used to indicate the significant difference (*P* < 0.05) among the five treatments within the same year after biochar application. Sobs: observed species, invsimpson: invers simpson

After one year of decomposition, the wood-inhabiting bacterial diversity in the 650 °C biochar treatment was significantly higher than the other treatments, respectively (*P* < 0.05) (Fig. 2a). This increased diversity relative to the other treatments persisted in the second year for the 650 °C /1.0 kg m^−2^ treatment. In the second year, differences also emerged in the 500 °C treatments, with increased diversity found with the 1.0 kg m^−2^ biochar treatment (*P* < 0.05) (Fig. 2a). The bacterial species richness did not differ between treatments during the decomposition period (Fig. 2b). After one-year decomposition, the bacterial evenness in the 500 °C biochar treatments was significantly lower than that in the control (*P* < 0.05) (Fig. 2c), but no differences were found among the treatments in the second year. Multivariate analysis of variance showed that the pyrolysis temperature, wood decomposition time, and the interaction between the pyrolysis temperature and the amount of biochar significantly affected the bacterial diversity (Additional file 1: Tables S2, S3).

### Wood-inhabiting bacterial community structure at the taxonomic level in different biochar treatments

**Fig. 3:**
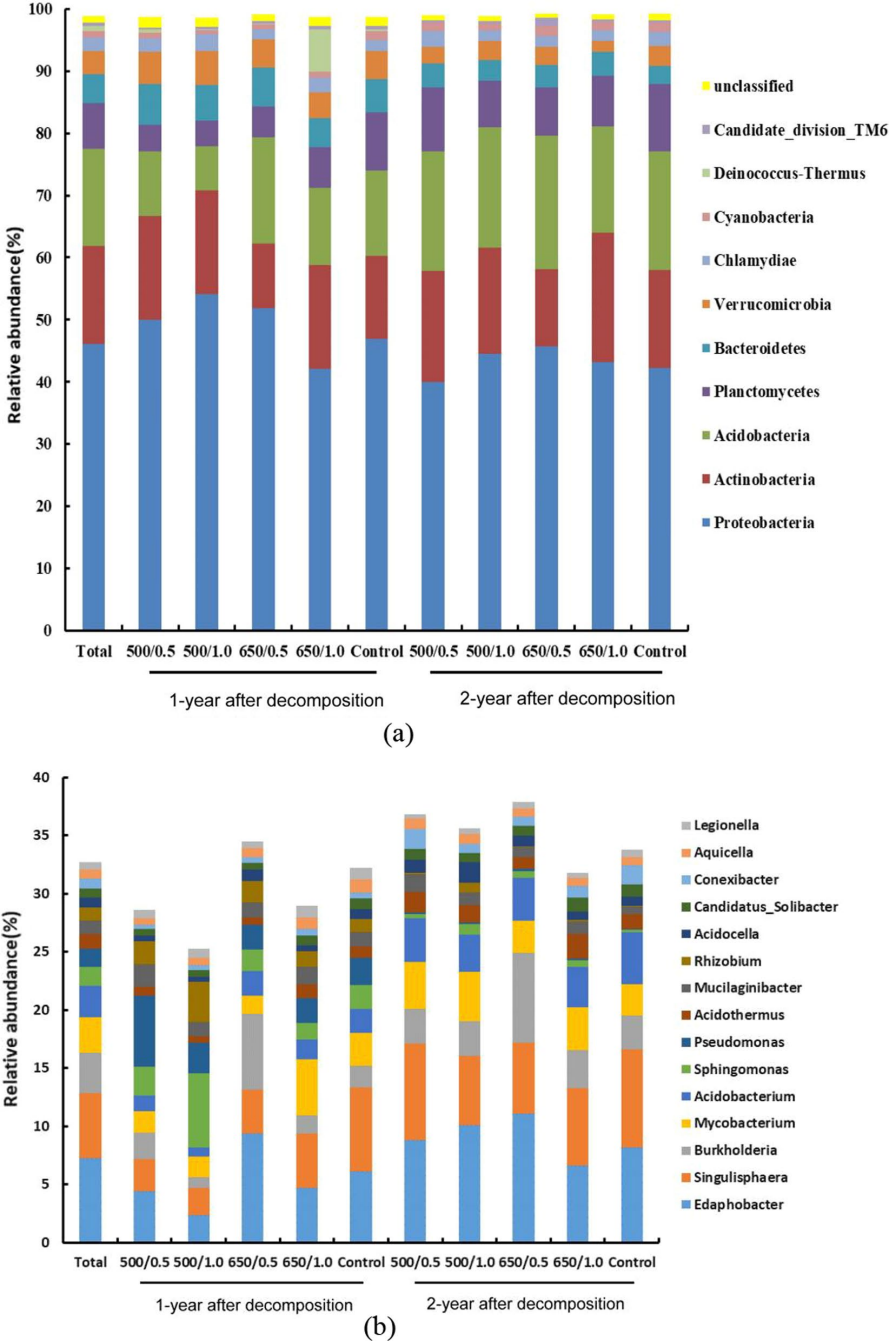
The relative abundance of wood-inhabiting bacteria at the phylum level (**a**) and the top 10 most abundant genera (**b**) after one and two years of decomposition in different biochar treatments. The number before and after "/" represents the pyrolysis temperature of biochar (°C) and application amount of biochar (kg m^−2^)

All sequences were divided into bacterial domains, and assigned to 16 141 OTUs, of which 98.9% of sequences were classified into 29 bacterial phyla and 445 bacterial genera. Proteobacteria (46.1%) was the most abundant phylum, followed by Actinobacteria (15.8%), Acidobacteria (15.7%), Planctomycetes (7.4%), Bacteroidetes (4.6%), and Verrucomicrobia (3.8%) (Fig. 3a).

After one year of decomposition, under the treatment with 1.0 kg m^−2^ biochar, the treatments with biochar produced at 500 °C had significantly lower abundance of Acidobacteria and Planctomycetes(7.17% and 4.07%), than the control (13.78 and 9.30%) (*P* < 0.05). And the abundance of Acidobacteria on the areas with the biochar applying amount of 1.0 kg m^−2^, more decreased in the treatment with biochar produced at 500 °C (7.17%) compared to that at 650 °C (12.31%). With the biochar produced at 650 °C, the treatment with 1.0 kg m^−2^ (16.81%) biochar had a higher abundance of Actinobacteria than that of the 0.5 kg m^−2^ (10.43%) (*P* < 0.05). For the 0.5 kg m^−2^ biochar application, the treatment with biochar produced at 650 °C (17.00%) increased the abundance of Acidobacteria compared to that produced at 500 °C (10.43%) (*P* < 0.05), while the abundance of Actinobacteria (10.43%) was lower than that at 500 °C (16.61%) (*P* < 0.05). For the biochar produced at 650 °C, the 1.0 kg m^−2^ treatment (16.81%) increased the abundance of Actinobacteria more than the 0.5 kg m^−2^ treatment (10.43%) (*P* < 0.05), and the abundance of Actinobacteria showed the same trend in the second year. The abundance of Proteobacteria in the treatments with biochar produced at 500 °C (0.5 kg m^−2^ and 1.0 kg m^−2^, 39.97% and 44.49%) decreased significantly in the second year compared to the first year (0.5 kg m^−2^ and 1.0 kg m^−2^, 50.01% and 54.13%) (*P* < 0.05). The detailed data on the relative abundance of the phyla (top 10) wood-inhabiting bacterial communities were listed in Additional file 1: Table S4.

The abundant genera included *Edaphobacter* (7.2%), *Singulisphaera* (5.6%), *Burkholderia* (3.4%), *Mycobacterium* (3.1%), *Acidobacterium* (2.7%), *Sphingomonas* (1.7%), *Pseudomonas* (1.6%)*, Acidothermus* (1.2%), *Mucilaginibacter* (1.2%), and *Rhizobium* (1.1%) (Fig. 3b). After one year of decomposition, in the treatments with biochar produced at 650 °C, the abundance of *Edaphobacter* were significantly higher in application with the amount of 0.5 kg m^−2^ (9.41%) than that of the control (6.14%) and other biochar treatments (500 °C/0.5 kg m^−2^, 500 °C/1.0 kg m^−2^and 650 °C/1.0 kg m^−2^, 4.41%, 2.40% and 4.68%) (*P* < 0.05). With the application amount of 1.0 kg m^−2^, the abundance of *Sphingomonas* and *Corynebacterinea* were significantly increased in the treatment with biochar produced at 650 °C (4.71% and 5.04%) compared to that produced at 500 °C (2.32% and 1.83%) (*P* < 0.05), while that the abundance of *Burkholderia* and *Sphingomonas* were significantly lower than that the treatment at 500 °C (6.56% and 6.33%) (*P* < 0.05).

After two years of decomposition, with the biochar produced at 650 °C, the abundance of *Acidothermus* was significantly higher in the 0.5 kg m^−2^ treatment (3.81%) than in the 1.0 kg m^−2^ treatment (3.16%) (*P* < 0.05), while that the abundance of *Sphingomonas* were significantly lower than that than in the 1.0 kg m^−2^ treatment (0.97%) (*P* < 0.05). With 1.0 kg m^−2^ application, the abundance of *Burkholderia* was significantly increased with biochar produced at 650 °C (5.26%) compared to 500 °C (1.99%) (*P* < 0.05). The detailed data on the relative abundance of the genera (top 15) wood-inhabiting bacterial communities were listed in Additional file 1: Table S4.

### Wood-inhabiting bacterial community structure at the OTU level in different biochar treatments

**Fig. 4:**
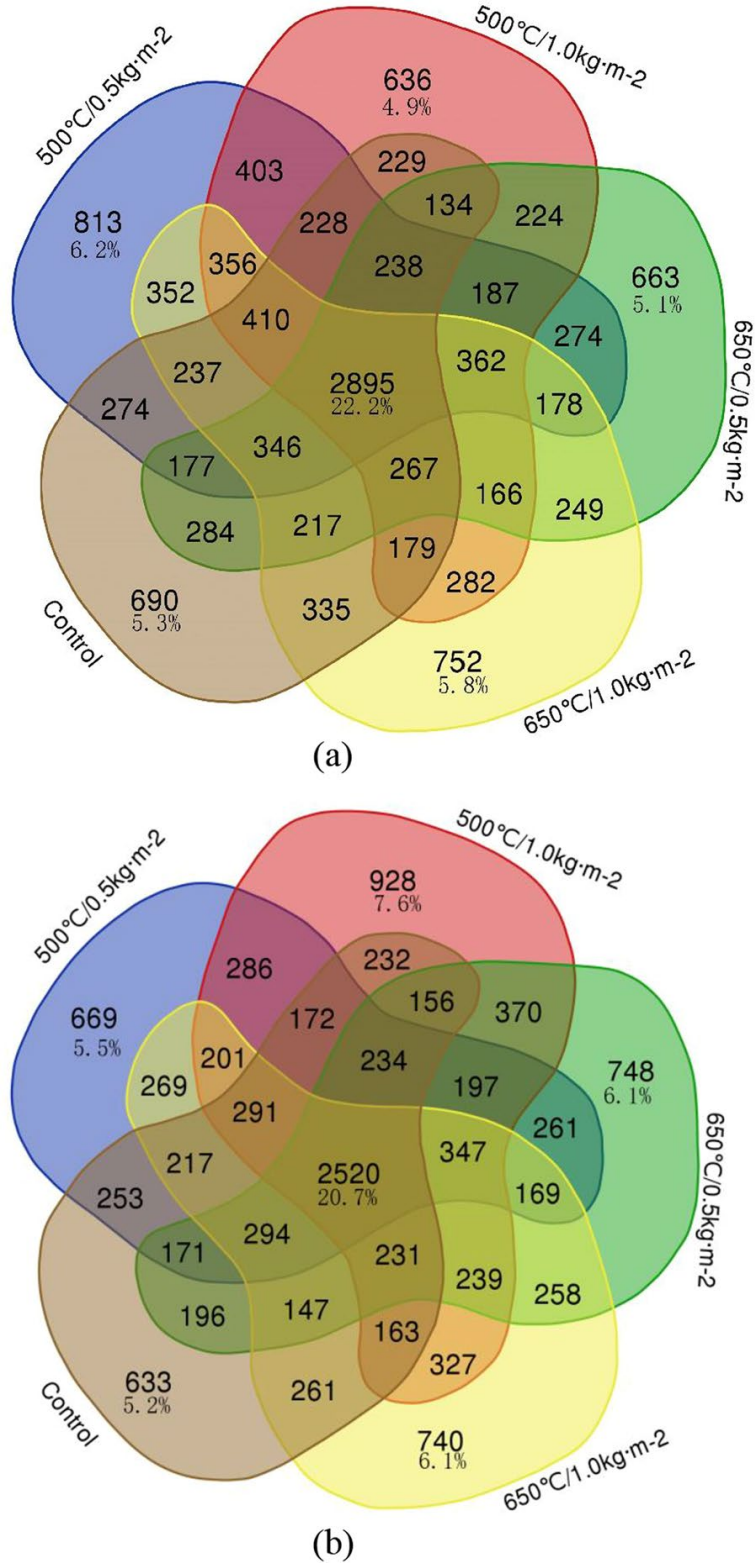
Venn diagram showing the wood-inhabiting bacteria unique and shared OTUs between different biochar treatments after one (**a**) and two years (**b**) of decomposition. The number before and after "/" represents the pyrolysis temperature of biochar (°C) and application amount of biochar (kg m^−2^)

The unique and shared OTUs between different treatments are shown in Fig. 4, demonstrating that 22.2% and 20.7% of the OTUs were shared among the biochar treatments in the first and second year, respectively. The number of shared and unique OTUs in different biochar treatments showed a similar pattern in the first and second year. The number of unique OTUs in biochar treatments increased with the increase of decomposition time, except with the 500 °C/0.5 kg m^−2^ treatment (Fig. 4).

**Fig. 5:**
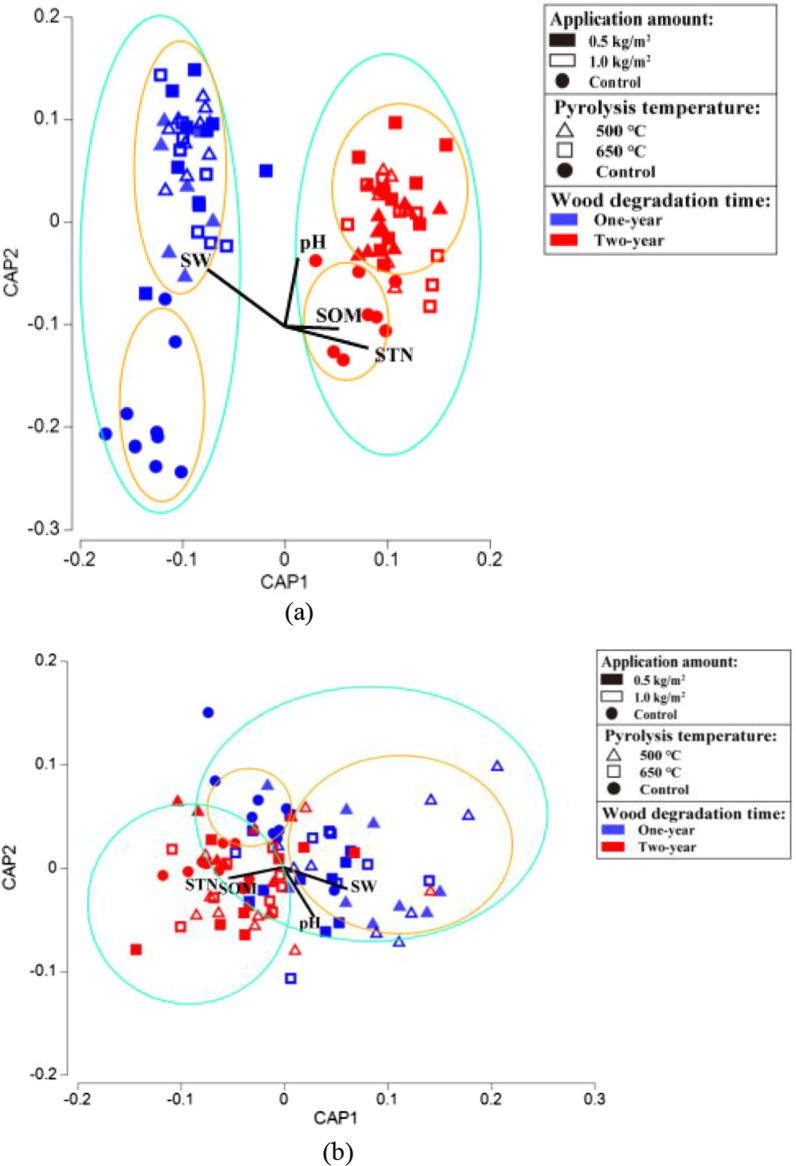
Distance Based Linear Model (DistLM) showing the **a** wood-inhabiting bacterial community structure and **b** functional structure using environmental factors as explanatory variables after one and two years of decomposition in different biochar treatments. The significance of the green circled areas different bacterial community structures and functional groups were formed in different years of wood degradation; the significance of the yellow circled areas different bacterial community structures and functional groups were formed between biochar treatment and blank control in the same year of wood degradation

CAP analysis based on the OTU data showed that the biochar treatments and the control formed distinct wood-inhabiting bacteria communities (*P* < 0.05) in both the first and second year (Fig. 5a). However, the treatments with biochar produced at different temperatures and with different biochar amounts did not form separate bacterial communities. Moreover, the wood-inhabiting bacterial community also differed between the first and second year. The difference in bacterial community structures was confirmed by PERMANOVA (Table 1). The DistLM using measured soil parameters as explanatory variables showed that the soil pH, soil organic matter (SOM), and soil total nitrogen (STN) were positively correlated with the bacterial communities in the second year (*P* < 0.05), in which the soil pH was correlated with the biochar treatment, while the SOM and STN were correlated with the control. The soil water content (SW) was positively correlated with the bacterial communities of biochar treatments in the first year (*P* < 0.05) (Fig. 5a, Additional file 1: Table S5).

**Table 1:** PERMANOVA showing the differences in wood-inhabiting bacteria community and functional structure in different biochar treatments

Group comparison		Community structure			Functional structure		
		t	P (perm)	Unique perms	t	P (perm)	Unique perms
Year1, Year2		3.3228	**0.001**	998	2.6443	**0.001**	999
Biochar treatment, control (Y1)		1.3463	**0.037**	999	1.713	**0.015**	999
Biochar treatment, control (Y2)		1.1141	**0.054**	999	0.99843	0.388	999
0.5 kg m^−2^, 1.0 kg m^−2^ (500 °C)	Y1	1.1698	0.128	983	0.64518	0.905	984
0.5 kg m^−2^, 1.0 kg m^−2^ (650 °C)		1.2098	0.085	975	1.5426	**0.051**	979
500 °C, 650 °C (0.5 kg m^−2^)		1.0955	0.239	984	0.98662	0.421	980
500 °C, 650 °C (1.0 kg m^−2^)		1.6486	**0.011**	973	1.1047	0.293	979
0.5 kg m^−2^, 1.0 kg m^−2^ (500 °C)	Y2	0.92382	0.627	979	0.9129	0.512	981
0.5 kg m^−2^, 1.0 kg m^−2^ (650 °C)		1.08	0.237	973	1.1278	0.263	972
500 °C, 650 °C (0.5 kg m^−2^)		1.0975	0.201	974	0.91339	0.536	978
500 °C, 650 °C (1.0 kg m^−2^)		0.8918	0.707	984	0.51887	0.946	980

The bold indicates the significant difference level at 95% and 99%, respectively

### Wood-inhabiting bacterial community structure of the predicted function in different biochar treatments

**Fig. 6:**
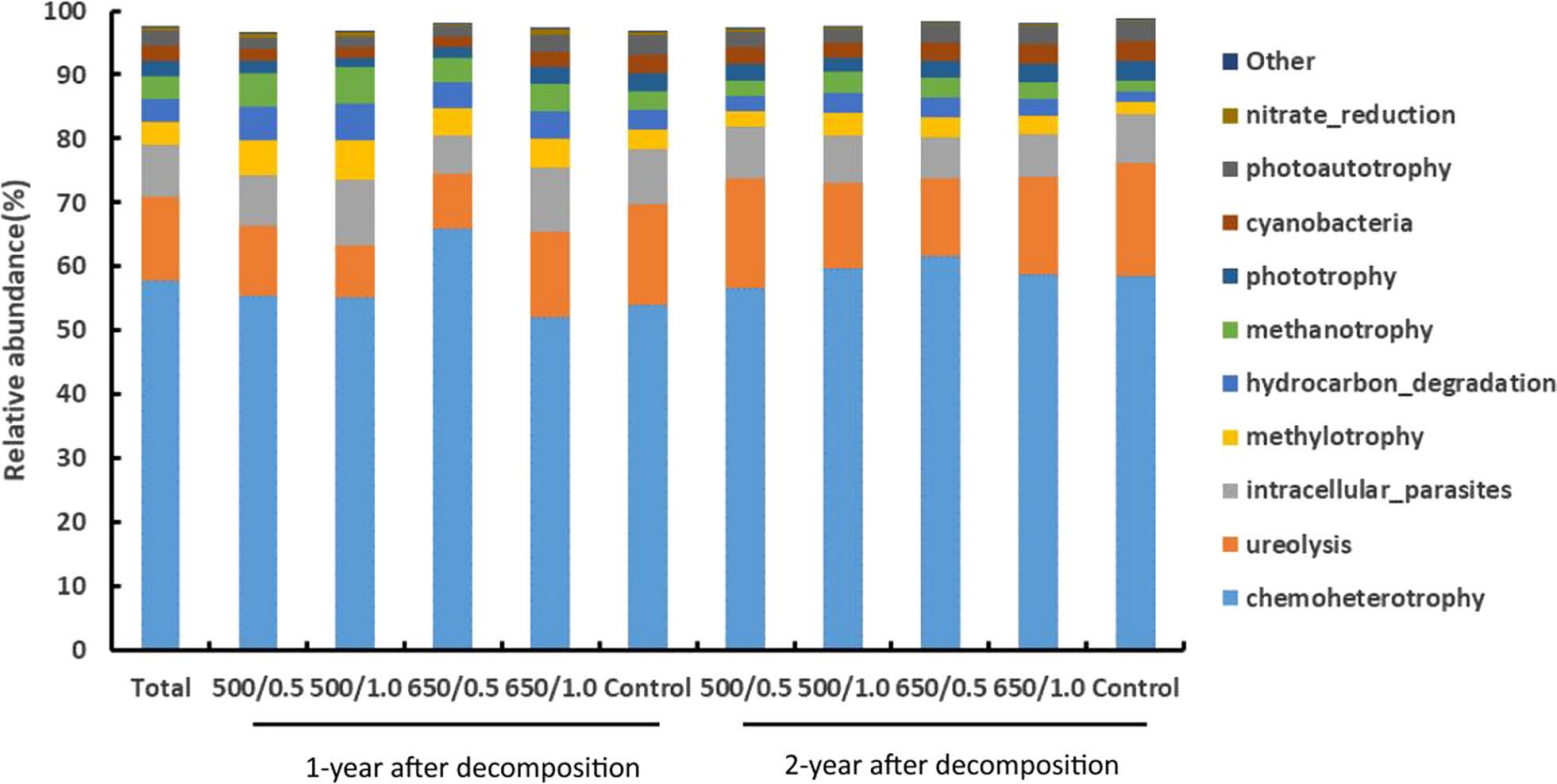
The most abundant functional groups of wood-inhabiting bacteria after one and two years of decomposition in different biochar treatments. The number before and after "/" represents the pyrolysis temperature of biochar (°C) and application amount of biochar (kg m^−2^), respectively

3558 OTUs (25.7% of the total OTUs) were assigned to 39 functional groups using FAPROTAX. Chemoheterotrophy (57.9%) was the most abundant functional group, followed by ureolysis (13.2%), intracellular parasites (7.9%), methylotrophy (3.7%), hydrocarbon degradation (3.5%), methanotrophy (3.5%), phototrophy (2.4%), and cyanobacteria (2.4%) (Fig. 6).

**Fig. 7:**
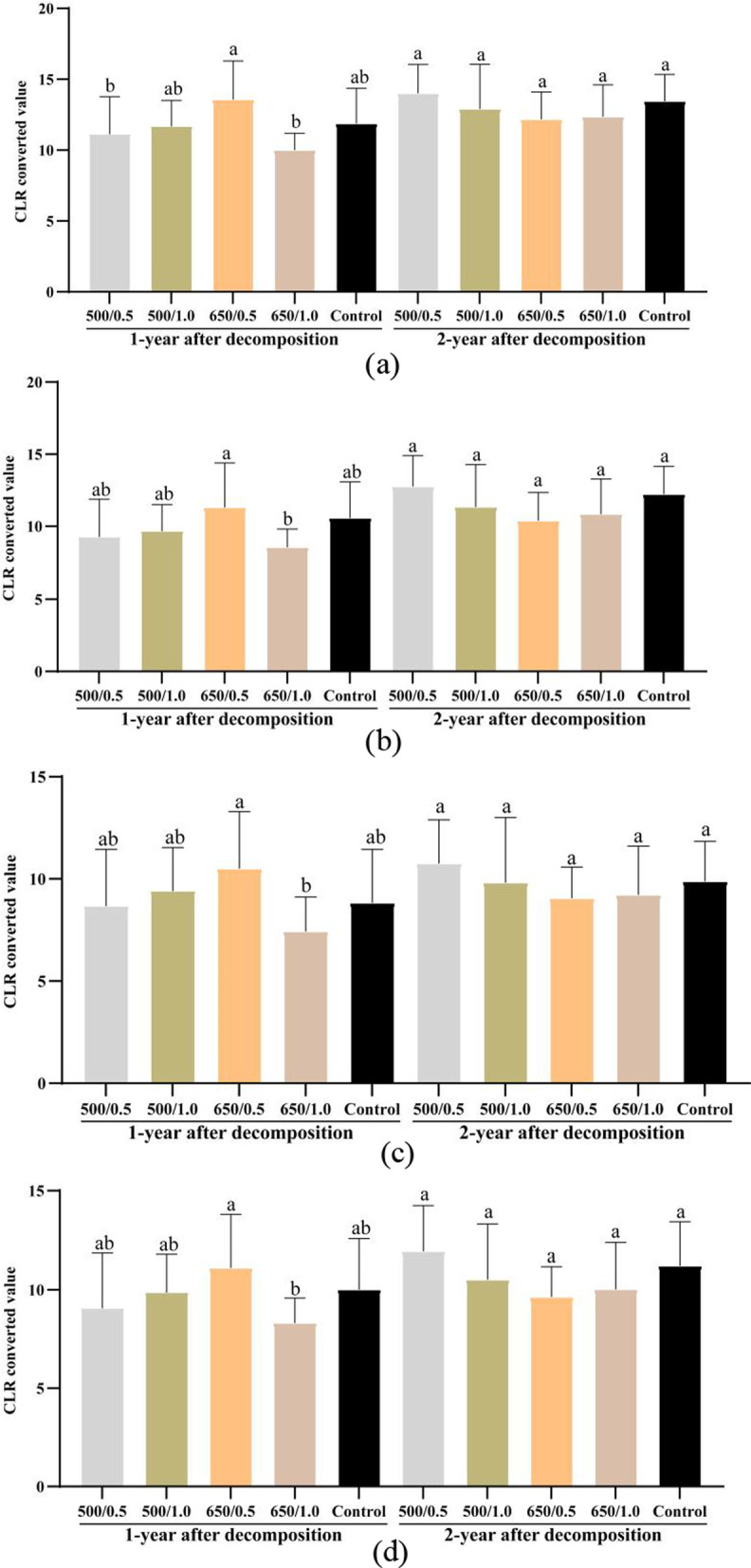
Centered Log-Ratio (CLR) transformation converted value of functional groups of wood-inhabiting bacteria (**a**) chemoheterotrophy (**b**) ureolysis (**c**) methylotrophy (**d**) intracellular parasites in different treatments, Lowercase letters were used to indicate the significant difference (*P* < 0.05) among the five treatments within the same year after wood decomposition. The number before and after "/" represents the pyrolysis temperature of biochar (°C) and applying amount of biochar (kg m^−2^), respectively

After one year of decomposition, with 0.5 kg m^−2^ biochar application amount, the abundance of chemoheterotrophy increased significantly in the treatment with biochar produced at 650 °C (65.88%) than in that produced at 500 °C (55.55%) (Fig. 7a). The treatment with biochar produced at 650 °C, the abundance of ureolysis, methylotrophy and intracellular parasites (13.20%, 4.41% and 10.11%) in the biochar application amount of 1.0 kg m^−2^ were significantly higher than that of 0.5 kg m^−2^ (8.53%, 4.22% and 6.09%) (Fig. 7b–d), while the abundance of chemoheterotrophy (52.21%) was significantly lower than that of 0.5 kg m^−2^ treatment (65.88%) (*P* < 0.05) (Fig. 7a).

After two years of decomposition, no significant effect were observed among biochar treatments with different pyrolysis temperatures and the amount of biochar different on the abundance of functional groups (Fig. 7a–d). The detailed data on the relative abundance of the function (top 10) of wood-inhabiting bacterial communities were listed in Additional file 1: Table S6. CAP analysis based on the functional data showed that the biochar treatments and the control formed distinct bacterial functional structures in the first year (*P* < 0.05), but not in the second year (Fig. 5b). Moreover, the bacterial functional structure also differed between the first and second year. Subsequent PERMANOVA confirmed the differences in community structures (Table 1). Similarly, the soil total nitrogen (STN) and soil organic matter (SOM) were positively correlated with bacterial functional structures in the second year (*P* < 0.05). The soil pH and soil water content (SW) were positively correlated with bacterial functional structures of the biochar treatments in the first year (*P* < 0.05) (Fig. 5b, Additional file 1: Table S5).

## Discussion

In this study, the wood-inhabiting bacterial structure and function during the wood decomposition under different biochar treatments were studied. Treatments with biochar produced at 650 °C had higher bacterial diversity than did those with biochar produced at 500 °C. Biochar has the characteristics of high carbon content, high pH, and high porosity, which can change the physical and chemical properties of soil after application, such as the soil nutrients and soil pH [2, 39]. One of the most important elements determining the pH and surface area of biochar is the pyrolysis temperature, through which the soil CO_2_ emissions, including microbial and root respiration, are affected [2, 40–42]. The biochar produced at high temperatures had a higher pH [29], which might increase the pH value of the soil, benefiting most soil bacteria [40, 43, 44]. A previous study has shown that biochar mixed with soil can significantly increase the soil pH [44]. In our study, the biochar was spread on the top of the vegetation rather than mixed with the soil directly, to avoid soil disturbance. This might partly explain some of the observations in our study in that although the soil pH in all the biochar treatments increased, only one biochar treatment of 500 °C/1.0 kg m^−2^ showed a significantly higher soil pH compared to the control.

The treatments with biochar produced at different temperatures and different application amounts significantly affected the abundance of certain wood-inhabiting bacterial taxa during the decomposition process, e.g., Acidobacteria and Actinobacteria. Acidobacteria can spread widely in various environments (i.e., the ocean and activated sludge), demonstrating general adaptability and functional diversity [45]. In this study, one year after beginning decomposition, the abundance of Acidobacteria in the areas with a biochar application amount of 1.0 kg m^−2^ decreased more in the treatment with biochar produced at 500 °C (7.17%) compared to 650 °C (12.31%). A previous study showed that soil pH may become a limiting factor for soil microbial growth following biochar application [46], In this study, the soil pH was significantly higher in the 500 °C/1.0 kg m^−2^ treatment than the other treatments (Additional file 1: Table S1), moreover, the relative abundance of Acidobacteria was negatively correlated with soil pH in the 500/1.0 treatment (Additional file 1: Table S7), which might affect the abundance of Acidobacteria. In addition, Acidobacteria from soil produce a higher amount of lignin decomposing enzyme, which may contribute to the degradation of polysaccharides in wood [47]. Most members of the Actinobacteria family can be associated with nutrient cycling, and can degrade cellulose and chitin as the main resource for the soil nutrient supply [48]. The abundance of Actinobacteria was significantly higher in the 1.0 kg m^−2^ treatment (16.81%) than in the 0.5 kg m^−2^ treatment (10.43%) with biochar produced at 650 °C. Actinobacteria can produce a series of extracellular enzymes to effectively decompose complex aromatic substances [49]. In this study, at pyrolysis temperature of 650 °C, under the high application amount of biochar, the relative abundance of Actinobacteria positively was correlated with soil pH, which promoted the growth of Actinobacteria (Additional file 1: Table S7). Li et al. also showed that biochar applied at both 2 kg m^−2^ and 6 kg m^−2^ to the soil in a China fir plantation significantly increased the proportion of Actinobacteria 90 days after application [50].

The treatments with 1.0 kg m^−2^ biochar, and the treatments with biochar produced at 650 °C (4.71%) significantly increased the relative abundance of *Sphingomonas* after one year of decomposition, compared to that produced at 500 °C (2.32%) after one year decomposition. Members of the genus *Sphingomonas* have an aerobic heterotrophic soil-based lifestyle, with an additional ability to degrade extraordinarily recalcitrant carbon sources and to produce related exopolysaccharides [51]. In the early decomposition process of wood, *Sphingomonas* is a common genus that uses simple carbon compounds [52]. The biochar produced by pyrolysis at a high temperature is rich in ash content [53], in this study, in the treatment with 650 °C/1.08 kg m^−2^, the abundance of *Sphingomonas* was positively correlated with soil organic matter, which promoted the growth of *Sphingomonas* (Additional file 1: Table S7). *Burkholderia* has the ability to degrade recalcitrant xenobiotics [54]. With the 1.0 kg m^−2^ biochar treatment, the abundance of *Burkholderia* significantly increased in the treatment with biochar produced at 650 °C (5.26%) compared to that produced at 500 °C (1.99%) after two years of decomposition. *Burkholderia* can be involved in the decay of forest litter in peat forest soil [12]. With increasing pyrolysis temperature of the biochar, the content of aromatic carbon in the biochar increases, as does the content of refractory carbon [18]. In this study, there is a close positive correlation between the abundance of *Burkholderia* and ureolysis with 650 °C/1.0 kg m^−2^ (Additional file 1: Table S7). Moreover, Fraver et al. showed that the most of the wood structures in the late stage of decomposition have obviously disintegrated and more recalcitrant substances remain [55], which might contribute to the increase in the abundance of certain specialized microbes.

The biochar treatments formed distinct wood-inhabiting bacterial communities during the decomposition process. Bacteria are more sensitive to unstable substrates and unstable carbon in biochar can directly affect the growth of bacteria [56]. The wood-inhabiting bacteria structure also differed during the decomposition period. This is consistent with other studies, showing that the microbial community structure changes as wood decomposition proceeds [57]. At the early stage of wood degradation, bacteria can use some easily available substances, such as polysaccharides. The wood components change with increasing degradation time, driving a shift in the microbial community [17, 58]. In addition, fresh biochar increases the activity of soil microorganisms and stimulate the decomposition of wood due to the labile carbon components on its surface [8, 44].

Similar to the bacterial community structure, the bacterial functional structure differed between the biochar treatments and the control in the first year of decomposition, but not in the second year. The change of bacterial community structure can reflect the change of bacterial community function to a certain extent, but in terms of the microbial functional redundancy, this is not always the case. Moreover, in our case, the functional analysis was based only on a small portion of the OTUs data, which may not reflect the true situation. Biochar can change the composition of understory vegetation, especially when mixed with soil [6]. The treatment with biochar produced at 650 °C, and 1.0 kg m^−2^ biochar application amount the abundance of methylotrophy (4.41%) were significantly higher than that in the 0.5 kg m^−2^ treatment (4.22%) The functional of methylotrophy can directly reflect the ability of bacteria degrade carbon-related organic matters. [59]. Biochar application can affect microbes directly by providing a large amount of carbon, or indirectly [2, 60]. At higher pyrolysis temperature and higher application rate, the abundance of microorganisms related to carbon cycle, such as methyl nutrition groups, was increased, thus improving the nutrient cycle efficiency and accelerating the process of carbon cycle [60].

Both biochar application and wood decomposition are long and complicated processes, and in this study, we only monitored the situation for two years after biochar application. In addition, fungi also contribute significantly to litter degradation, and the interaction between microbes during wood degradation is important. Therefore, longer term monitoring is needed to understand the full picture of wood-inhabiting microbes during wood decomposition after biochar application in boreal forests.

## Conclusion

The interaction between the biochar pyrolysis temperature and the amount of biochar applied significantly affected the wood-inhabiting bacterial diversity (*P* < 0.05). With the extension of the decomposition time, the wood-inhabiting bacterial diversity and species richness decreased. Biochar application shifted the wood-inhabiting bacterial community during the wood decomposition process, despite the different biochar pyrolysis temperatures and application amounts. Similarly, biochar application shifted the wood-inhabiting bacterial function in the first year of degradation. Proteobacteria, Actinobacteria, Acidobacteria, *Edaphobacter, Singulisphaera,* and *Burkholderia* were the most abundant wood-inhabiting bacterial taxa after biochar application, and the abundance was affected by the biochar pyrolysis temperature and application amount during the decomposition process. Long-term monitoring is needed to better understand the effects of biochar application on the wood-inhabiting microbial community in boreal forests.

## Supplementary Information

**Additional file 1: Table S1** The soil physical chemical properties under different biochar treatments. **Table S2** Multivariate analysis of variance showing the difference in wood loss rate, bacterial community diversity, richness and evenness under different biochar treatments by using the pyrolysis temperatures, applying amounts and wood degradation time since application as variates. **Table S3** Difference of application amount of biochar at the same pyrolysis temperature on diversity of bacteria in the same degradation year. **Table S4** The relative abundance (%) of the phyla (top 10) and the genera (top 15) of wood-inhabiting bacteria in different biochar treatments. **Table S5** DistLM showing the correlation between the soil properties and the wood-inhabiting bacterial community and functional structures. **Table S6** The relative abundance (%) of the function (top 10) of wood-inhabiting bacteria in different biochar treatments. **Table S7** Correlation between relative abundance (%) of Acidobacteria, Actinobacteria, *Singulisphaera*, *Burkholderia* and wood degradation rate, soil physical and chemical properties and some functions during wood degradation under different biochar treatments.

## Data Availability

The datasets generated during and/or analyzed during the current study are available in the NCBI Sequence Read Archive (SRA) database (https://www.ncbi.nlm.nih.gov/sra) with the following accession number: PRJNA797190. The material is original and the manuscript has not been submitted for publication elsewhere while under consideration for publication in Environmental Microbiome, and has not been previously published.
