# Supplementary material for: The impact of biochar on wood-inhabiting bacterial community and its function in a boreal pine forest
Source: Environ Microbiome. 2022 Aug 30;17:45. doi: 10.1186/s40793-022-00439-9 (PMC9429645; doi:10.1186/s40793-022-00439-9)
Supplement: Supplementary file 1 — Additional file 1: Table S1 The soil physical chemical properties under different biochar treatments. Table S2 Multivariate analysis of variance showing the difference in wood loss rate, bacterial community diversity, richness and evenness under different biochar treatments by using the pyrolysis temperatures, applying amounts and wood degradation time since application as variates. Table S3 Difference of application amount of biochar at the same pyrolysis temperature on diversity of bacteria in the same degradation year. Table S4 The relative abundance (%) of the phyla (top 10) and the genera (top 15) of wood-inhabiting bacteria in different biochar treatments. Table S5 DistLM showing the correlation between the soil properties and the wood-inhabiting bacterial community and functional structures. Table S6 The relative abundance (%) of the function (top 10) of wood-inhabiting bacteria in different biochar treatments. Table S7 Correlation between relative abundance (%) of Acidobacteria, Actinobacteria, Singulisphaera, Burkholderia and wood degradation rate, soil physical and chemical properties and some functions during wood degradation under different biochar treatments. [file 40793_2022_439_MOESM1_ESM.docx]

**Supplementary files**

**Table S1.** The soil physical chemical properties under different biochar treatments

| Degradation time | treatment | Soil water content (%) | Soil pH | Soil organic matter (g/kg) | Soil total nitrogen(g/kg) |
| --- | --- | --- | --- | --- | --- |
| 1-year after | 500 ℃/ 0.5 kg·m⁻² | 57.77±11.51 | 5.17±0.2 | 209.97±68.05 | 16.33±2.56 |
|  | 500 ℃/ 1.0 kg·m⁻² | 60.09±1.4 | **5.64±0.21*** | 186.67±20.3 | 17.31±6.03 |
|  | 650 ℃/ 0.5 kg·m⁻² | 65.5±5.22 | 5.24±0.26 | 214.88±19.78 | 16.98±7.1 |
|  | 650℃/ 1.0 kg·m⁻² | 63.64±10.21 | 5.25±0.29 | 214.02±125.18 | 15.21±1.16 |
|  | Control | 52.64±9.19 | 4.91±0.19 | 198.77±34.4 | 16.69±2.28 |
| 2-year after | 500 ℃/ 0.5 kg·m⁻² | 55.44±2.66 | 5.12±0.11 | 206.95±59.01 | 17.27±2.11 |
|  | 500 ℃/ 1.0 kg·m⁻² | 50.45±4.41 | **5.61±0.45*** | 221.48±42.75 | 18.74±4.52 |
|  | 650 ℃/ 0.5 kg·m⁻² | 53.96±2.74 | 5.31±0.38 | 207.73±63.91 | 16.28±6.17 |
|  | 650℃/ 1.0 kg·m⁻² | 53.92±4.83 | 5.31±0.27 | 233.38±47.66 | 15.77±0.74 |
|  | Control | 56.95±7.08 | 4.86±0.03 | 226.13±22.39 | 16.39±0.82 |

The values are shown as means ± standard deviation (n = 9). **P* < 0.05.

**Table S2.** Multivariate analysis of variance showing the difference in wood loss rate , bacterial community diversity, richness and evenness under different biochar treatments by using the pyrolysis temperatures, applying amounts and wood degradation time since application as variates

| Variate | wood loss mass rate | | Species richness (sobs) | | Evenness  (simpsoneven) | | α-diversity(invsimpson) | | |
| --- | --- | --- | --- | --- | --- | --- | --- | --- | --- |
|  | *F*-value | *P*-value | *F*-value | *P*-value | *F*-value | *P*-value | *F*-value | *P*-value | |
| Pyrolysis temperature | 0.4626 | 0.4984 | 0.2022 | 0.6543 | 0.3797 | 0.5395 | 41.6428 | **<0.01** |  |
| Applying amount | 0.7977 | 0.3745 | 0.9392 | 0.3358 | 0.7820 | 0.3792 | 0.8434 | 0.3612 | |
| Wood degradation time | 20.5445 | **<0.01** | 23.3765 | **<0.01** | 1.0867 | 0.3004 | 10.3628 | **<0.01** | |
| Temperature×Amount | 0.0732 | 0.7874 | 3.5618 | 0.0632 | 0.6143 | 0.4355 | 6.0933 | **<0.05** | |
| Temperature×Time | 0.9354 | 0.3364 | 0.0181 | 0.8933 | 2.3373 | 0.1303 | 2.4561 | 0.1210 | |
| Amount×Time | 0.2862 | 0.5942 | 0.2651 | 0.6082 | 3.7487 | 0.0564 | 2.0663 | 0.1545 | |
| Temperature×Amount×Time | 0.0699 | 0.7922 | 4.9473 | **<0.05** | 0.0326 | 0.8572 | 0.5196 | 0.4731 | |

**Table S3.** Difference of application amount of biochar at the same pyrolysis temperature on diversity of bacteria in the same degradation year

| Variate | | One year after decomposition | Two year after decomposition |
| --- | --- | --- | --- |
| 500℃ | 0.5kg·m⁻² | 62.76±7.18a | 43.81±4.52b |
|  | 1.0kg·m⁻² | 60.16±7.19a | 59.64±3.51a |
|  | Control | 66.31±6.79a | 62.80±5.61a |
|  |  |  |  |
| 650℃ | 0.5kg·m⁻² | 104.46±5.83a | 78.28±5.70a |
|  | 1.0kg·m⁻² | 86.94±6.96a | 66.88±5.90a |
|  | Control | 66.31±6.79b | 62.80±5.61a |

The values are shown as means ± standard deviation (n = 9). Lowercase letters were used to indicate the significant difference (*P* < 0.05) among the three treatments within the same year after wood decomposition.

**Table S4** The relative abundance (%) of the phyla (top 10) and the genera (top 15) of wood-inhabiting bacteria in different biochar treatments

|  | Bacterial  community | Decomposition after one-year (%) | | | | | Decomposition after two-year (%) | | | | |
| --- | --- | --- | --- | --- | --- | --- | --- | --- | --- | --- | --- |
|  |  | 500 ℃/ 0.5kg·m⁻² | 500 ℃/ 1.0kg·m⁻² | 650 ℃/ 0.5kg·m⁻² | 650 ℃/ 1.0kg·m⁻² | Control | 500 ℃/ 0.5kg·m⁻² | 500 ℃/ 1.0kg·m⁻² | 650 ℃/ 0.5kg·m⁻² | 650 ℃/ 1.0kg·m⁻² | Control |
| phyla | Proteobacteria | 50.01±9.84a | 54.13±4.72a | 51.91±8.23a | 42.04±2.64a | 46.95±1.368a | 39.97±4.80a | 44.49±2.25a | 45.64±3.24a | 43.19±5.21a | 42.21±0.84a |
|  | Actinobacteria | 16.61±3.36a | 16.63±2.45a | 10.43±1.60b | 16.81±4.07a | 13.29±2.04ab | 17.85±4.15ab | 17.14±4.71ab | 12.42±0.95b | 20.80±2.65a | 15.83±2.69ab |
|  | Acidobacteria | 10.43±4.98bc | 7.17±0.88c | 17.00±3.79a | 12.31±2.76ab | 13.78±1.57ab | 19.34±3.94a | 19.32±6.17a | 21.56±2.01a | 17.16±2.40a | 19.11±3.40a |
|  | Planctomycetes | 4.26±1.91b | 4.07±0.88b | 5.02±3. 17ab | 6.66±2.32ab | 9.30±2.82a | 10.21±1.89a | 7.52±1.63a | 7.70±3.46a | 8.15±3.72a | 10.70±1.24a |
|  | Bacteroidetes | 6.55±1.13a | 5.83±1.55a | 6.26±1.98a | 4.61±1.06a | 5.33±0.86a | 3.94±1.76a | 3.29±1.02a | 3.61±1.09a | 3.79±1.66a | 3.00±1.19a |
|  | Verrucomicrobia | 5.25±0.45a | 5.46±1.07a | 4.54±2.05a | 4.11±0.81a | 4.64±1.00a | 2.66±0.79a | 3.16±1.59a | 2.97±1.28a | 1.81±0.72a | 3.18±1.48a |
|  | Chlamydiae | 2.17±1.01a | 2.58±1.39a | 1.57±0.92a | 2.40±0.98a | 1.76±0.27a | 2.49±1.36a | 1.69±0.88a | 1.75±0.15a | 1.73±0.18a | 2.26±1.16a |
|  | Cyanobacteria | 0.86±0.78a | 0.70±0.16a | 0.82±0.53a | 1.03±0.16a | 1.43±0.09a | 1.31±0.62a | 1.05±0.41a | 1.60±1.00a | 1.25±0.40a | 1.54±0.14a |
|  | Deinococcus  Thermus | 0.56±0.37a | 0.12±0.06a | 0.16±0.12a | 0.78±0.48a | 0.30±0.22a | 0.01±0.00a | 0.02±0.02a | 0.01±0.00a | 0.01±0.00a | 0.01±0.00a |
|  | Candidate division TM6 | 0.32±0.17a | 0.39±0.20a | 0.42±0.22a | 0.56±0.20a | 0.50±0.12a | 0.44±0.20a | 0.40±0.10a | 1.33±0.80a | 0.40±0.02a | 0.40±0.03a |
| genera | *Edaphobacter* | 4.41±1.71bc | 2.40±0.41c | 9.41±3.24a | 4.68±1.31bc | 6.14±0.65b | 8.80±5.09a | 10.07±6.65a | 11.11±5.19a | 6.65±2.20a | 8.20±3.72a |
|  | *Singulisphaera* | 2.80±1.68b | 2.32±0.61b | 3.71±2.54ab | 4.71±2.54a | 7.18±2.58a | 8.34±2.33a | 6.00±1.73a | 6.10±3.05a | 6.65±3.15a | 8.40±0.69a |
|  | *Burkholderia* | 2.26±1.69ab | 0.90±0.29b | 6.56±5.19a | 1.52±0.32b | 1.87±0.49b | 2.95±1.49b | 1.99±0.82b | 7.71±5.69a | 5.26±1.44a | 3.88±1.78ab |
|  | *Corynebacterineae* | 1.87±0.88b | 1.83±0.68b | 1.61±0.72b | 5.04±1.92a | 2.90±0.89b | 4.51±1.71a | 4.31±0.85a | 2.77±0.56a | 3.73±2.07a | 2.77±1.35a |
|  | *Micrococcineae* | 6.75±2.18a | 6.47±0.70a | 3.14±2.01b | 1.74±0.80b | 1.65±0.62b | 0.98±0.40a | 2.09±1.00a | 1.46±1.16a | 1.70±0.60a | 0.96±0.70a |
|  | *Acidimicrobineae* | 1.98±0.76a | 1.53±0.744a | 1.36±0.82a | 2.93±0.50a | 3.12±0.91a | 3.42±0.53a | 2.55±1.63a | 2.11±0.56a | 3.83±0.61a | 3.96±0.92a |
|  | *Acidobacterium* | 1.36±0.50a | 0.80±0.45a | 2.06±1.30a | 1.67±0.84a | 2.04±0.12a | 3.81±0.48a | 3.16±1.42b | 3.68±1.80ab | 3.44±0.84ab | 4.48±0.71a |
|  | *Sphingobacteriaceae* | 4.13±1.46a | 2.82±0.97a | 4.46±2.40a | 2.93±0.94a | 3.83±0.69a | 1.75±0.93a | 1.55±1.00a | 1.12±0.04a | 1.29±0.50a | 0.81±0.37a |
|  | *Sphingomonas* | 2.48±1.87b | 6.33±2.83a | 1.90±0.438b | 1.43±0.69b | 2.05±1.00b | 0. 35±0.31b | 0.97±0.47a | 0.61±0.30ab | 0.59±0. 29ab | 0.19±0.08b |
|  | *Pseudomonas* | 6.11±0.028a | 2.66±0.73a | 2.12±0.464a | 2.15±0.412a | 2.33±0.68 a | 0.10±0.001a | 0.14±0.05a | 0.18±0.07a | 0.16±0.04a | 0.06±0.01a |
|  | *Frankineae* | 1.01±0.51a | 1.15±0.37a | 0.91±0.48a | 1.55±0.41a | 1.14±0.46a | 1.97±0.73ab | 1.78±0.78ab | 1.33±0.06b | 2.40±0.18a | 1.35±0.30b |
|  | *Rhizobium* | 2.01±0.48 a | 3.51±1.48a | 1.85±1.41a | 1.32±0.49a | 0.18±0.56a | 0.13±0.01b | 0.85±0.50a | 0.12±0.08b | 0.10±0.03b | 0.09±0.05b |
|  | *Acidocella* | 0.51±0.27a | 0.41±0.20a | 0.99±0.54a | 0.49±0.12a | 0.79±0.07a | 1.13±0.68a | 1.72±0.93a | 0.93±0.16a | 0.76±0.38a | 0.81±0.28a |
|  | *Conexibacteraceae* | 0.005±0.002a | 0.004±0.001a | 0.010±0.003a | 0.005±0.001a | 0.008±0.000a | 0.017±0.002a | 0.007±0.001a | 0.008±0.002a | 0.010±0.002a | 0.017±0.005a |
|  | *Candidatus_Solibacter* | 0.58±0.20a | 0.55±0.20a | 0.60±0.03a | 0.82±0.14a | 0.96±0.18a | 0.94±0.48a | 0.82±0.15a | 0.83±0.11a | 1.16±0.39a | 1.02±0.39a |

The values are shown as means ± standard deviation (n = 9). Lowercase letters were used to indicate the significant difference (P < 0.05) among the five treatments within the same year after wood decomposition.

**Table S5.** DistLM showing the correlation between the soil properties and the wood-inhabiting bacterial community and functional structures

|  | Community structure | | | |  | Functional structure | | | | |
| --- | --- | --- | --- | --- | --- | --- | --- | --- | --- | --- |
| Soil property | SS(trace) | Pseudo-F | P | Prop. |  | | SS(trace) | Pseudo-F | P | Prop. |
| Soil water content | 9627.3 | 4.985 | **0.001** | 0.053611 |  | | 554.52 | 4.0289 | **0.003** | 0.043779 |
| Soil pH | 4143.7 | 2.0785 | **0.018** | 0.023075 |  | | 356.75 | 2.5503 | **0.032** | 0.028165 |
| Soil organic matter | 5141.9 | 2.594 | **0.006** | 0.028633 |  | | 261.63 | 1.856 | 0.096 | 0.020656 |
| Soil total nitrogen | 9127 | 4.7121 | **0.001** | 0.050825 |  | | 415.8 | 2.9868 | **0.014** | 0.032827 |

**Table S6** The relative abundance (%) of the function (top 10) of wood-inhabiting bacteria in different biochar treatments

| Bacterial  function | Decomposition after one-year (%) | | | | | Decomposition after two-year (%) | | | | | |
| --- | --- | --- | --- | --- | --- | --- | --- | --- | --- | --- | --- |
|  | 500 ℃/ 0.5kg·m⁻² | 500 ℃/ 1.0kg·m⁻² | 650 ℃/ 0.5kg·m⁻² | 650 ℃/ 1.0kg·m⁻² | Control | 500 ℃/ 0.5kg·m⁻² | 500 ℃/ 1.0kg·m⁻² | 650 ℃/ 0.5kg·m⁻² | 650 ℃/ 1.0kg·m⁻² | | Control |
| chemoheterotrophy | 55.55±14.07b | 55.30±10.52ab | 65.88±15.542a | 52.21±8.06b | 54.02±3.63ab | 56.69±7.56a | 59.78±13.12a | 61.70±13.30a | 58.91±8.03a | | 58.60±8.60a |
| ureolysis | 10.99±3.20ab | 8.01±1.08ab | 8.53±1.54a | 13.20±3.09b | 15.68±5.88ab | 17.19±5.84a | 13.38±5.78a | 12.22±5.84a | 15.07±8.16a | | 17.47±3.90a |
| Intracellular parasites | 7.82±4.08ab | 10.34±5.42ab | 6.09±2.73a | 10.11±3.23b | 8.68±1.96ab | 7.94±4.36a | 7.28±5.27a | 6.24±4.02a | 6.70±4.10a | | 7.85±6.52a |
| methylotrophy | 5.41±3.88ab | 6.08±2.25ab | 4.22±2.58a | 4.41±2.34b | 3.04±1.97ab | 2.47±1.38a | 3.54±1.44a | 3.24±1.06a | 2.82±1.41a | | 1.79±0.82a |
| Hydrocarbon degradation | 5.21±3.97ab | 5.69±2.37ab | 3.99±1.64a | 4.29±2.30b | 2.99±0.97ab | 2.39±1.39a | 3.21±2.37a | 3.03±1.89a | 2.66±1.40a | | 1.68±0.73a |
| methanotrophy | 5.21±3.97ab | 5.69±2.37ab | 3.99±1.64a | 4.29±2.30b | 2.99±0.97ab | 2.39±1.39a | 3.21±2.37a | 3.03±1.89a | 2.66±1.40a | | 1.68±0.73a |
| phototrophy | 1.90±0.60ab | 1.61±1.18ab | 1.64±1.13a | 2.60±1.18b | 2.90±1.13ab | 2.57±1.33a | 2.28±1.27a | 2.78±1.40a | | 2.93±1.11a | 3.06±0.92a |
| cyanobacteria | 1.86±0.80ab | 1.59±0.98ab | 1.63±1.14a | 2.57±1.15b | 2.89±1.12ab | 2.56±1.33a | 2.27±1.27a | 2.78±1.40a | | 2.92±1.11a | 3.06±0.92a |
| photoautotrophy | 1.86±0.80ab | 1.59±0.98ab | 1.63±1.14a | 2.57±1.15b | 2.89±1.12ab | 2.56±1.33a | 2.27±1.27a | 2.78±1.40a | 2.92±1.11a | | 3.06±0.92a |
| Nitrate reduction | 0.55±0.29ab | 0.79±0.62ab | 0.36±0.21a | 0.86±0.42b | 0.58±0.29ab | 0.40±0.19a | 0.27±0.16a | 0.32±0.14a | 0.37±0.15a | | 0.32±0.14a |

The values are shown as means ± standard deviation (n = 9). Lowercase letters were used to indicate the significant difference (P < 0.05) among the five treatments within the same year after wood decomposition under Centered Log-Ratio (CLR) transformation converted value.

**Table S7** Correlation between relative abundance (%) of Acidobacteria, Actinobacteria, *Singulisphaera* , *Burkholderia* and wood degradation rate, soil physical and chemical properties and some functions during wood degradation under different biochar treatments

|  | | treatment | Wood mass loss rate(%) | Soil organic matter (g/kg) | Soil water content (%) | Soil pH | Soil total nitrogen(g/kg) | chemoheterotrophy | ureolysis | methylotrophy | cellulolysis | Aromatic compound degradation |
| --- | --- | --- | --- | --- | --- | --- | --- | --- | --- | --- | --- | --- |
|  |  |  | r | r | r | r | r | r | r | r | r | r |
| Acidobacteria | 1-year after | 500/0.5 | -0.948 | -0.979 | -0.998 | 0.934 | -0.434 | -0.828 | -0.122 | -0.515 | -0.520 | -0.520 |
|  |  | 500/1.0 | -0.545 | -0.403 | -0.403 | -0.979 | 0.805 | -0.994 | 0.526 | 0.237 | -0.625 | -0.625 |
|  |  | 650/0.5 | 0.979 | 0.860 | -0.977 | 0.338 | 0.299 | -0.124 | 0.469 | -0.425 | -0.039 | -0.039 |
|  |  | 650/1.0 | -0.942 | 0.916 | -0.237 | -0.030 | 0.640 | 0.163 | 0.993 | -0.173 | -0.908 | -0.908 |
|  |  | Control | -0.783 | 0.735 | 0.165 | 0.988 | -0.994 | 0.184 | -0.997 | -0.536 | -0.991 | -0.991 |
|  | 2-year after | 500/0.5 | 0.582 | -0.558 | 0.101 | 0.997 | -0.934 | 0.992 | 0.128 | -0.912 | -1.000 | -1.000 |
|  |  | 500/1.0 | 0.368 | -0.046 | -0.977 | 0.817 | -0.776 | 0.913 | -0.866 | 0.828 | -0.999 | -0.999 |
|  |  | 650/0.5 | 0.867 | -0.390 | 0.812 | 0.621 | -0.893 | 0.711 | -0.890 | 0.537 | 0.563 | 0.563 |
|  |  | 650/1.0 | 0.575 | -0.477 | -0.513 | 0.047 | -0.533 | 0.897 | -0.993 | 0.653 | 0.992 | 0.992 |
|  |  | Control | 0.551 | -0.702 | 0.705 | -0.307 | 0.945 | 0.943 | -0.644 | -0.711 | 0.824 | 0.824 |
| Actinobacteria | 1-year after | 500/0.5 | 0.081 | -0.038 | -0.180 | -0.121 | 0.770 | -0.744 | 0.934 | -0.956 | 0.955 | 0.704 |
|  |  | 500/1.0 | -0.556 | -0.392 | -0.392 | -0.976 | 0.812 | -0.995 | 0.536 | 0.225 | -0.847 | -0.616 |
|  |  | 650/0.5 | -0.834 | -0.244 | 0.839 | -0.906 | 0.466 | -0.617 | 0.296 | 0.942 | 0.435 | -0.681 |
|  |  | 650/1.0 | 0.310 | -0.377 | -0.978 | .998 | -0.751 | -0.982 | -0.091 | 0.980 | -0.922 | -0.443 |
|  |  | Control | -0.888 | 0.590 | -0.029 | 0.938 | -0.953 | -0.010 | -0.994 | -0.689 | 0.117 | -0.998 |
|  | 2-year after | 500/0.5 | 0.737 | 0.891 | 0.975 | -0.195 | -0.242 | 0.005 | 0.969 | -0.297 | 0.014 | 0.149 |
|  |  | 500/1.0 | -0.657 | 0.376 | 0.851 | -0.963 | 0.941 | -0.997 | 0.983 | -0.968 | 0.972 | 0.926 |
|  |  | 650/0.5 | 0.986 | -0.686 | 0.964 | 0.310 | -0.680 | 0.911 | -0.993 | 0.797 | -0.566 | 0.816 |
|  |  | 650/1.0 | 0.748 | -0.819 | -0.794 | 0.998 | 0.901 | 0.337 | 0.229 | 0.678 | 0.720 | -0.235 |
|  |  | Control | -0.871 | 0.760 | 0.659 | -0.928 | 0.261 | 0.265 | 0.808 | -0.652 | 0.923 | -0.623 |
| *Singulisphaera* | 1-year after | 500/0.5 | -0.922 | -0.961 | -0.991 | 0.905 | -0.366 | -0.867 | -0.049 | -0.577 | 0.018 | -0.456 |
|  |  | 500/1.0 | -0.541 | -0.407 | -0.407 | -0.980 | 0.802 | -0.994 | 0.522 | 0.241 | -0.856 | -0.629 |
|  |  | 650/0.5 | 0.398 | 0.912 | -0.389 | -0.575 | 0.952 | -0.883 | 0.992 | 0.496 | -0.811 | -0.840 |
|  |  | 650/1.0 | -0.918 | 0.887 | -0.300 | 0.036 | 0.588 | 0.098 | 0.983 | -0.108 | -0.470 | -0.934 |
|  |  | Control | 0.879 | -0.605 | 0.009 | -0.945 | 0.959 | -0.010 | 0.996 | 0.675 | -0.137 | .999 |
|  | 2-year after | 500/0.5 | 0.970 | 0.568 | 0.963 | 0.296 | -0.674 | 0.480 | 0.970 | -0.716 | 0.489 | -0.340 |
|  |  | 500/1.0 | -0.940 | 0.779 | 0.489 | -0.973 | 0.987 | -0.911 | 0.948 | -0.968 | 0.736 | 0.627 |
|  |  | 650/0.5 | -0.993 | 0.862 | -1.000 | -0.031 | 0.448 | -0.990 | 0.986 | -0.934 | 0.312 | -0.945 |
|  |  | 650/1.0 | -0.496 | 0.393 | 0.431 | -0.140 | 0.610 | -0.851 | 1.000 | -0.579 | -0.530 | -1.000 |
| *Burkholderia* |  | Control | -0.946 | 0.866 | 0.510 | -0.844 | 0.080 | 0.084 | 0.902 | -0.502 | 0.838 | -0.756 |
|  | 1-year after | 500/0.5 | -0.376 | -0.263 | -0.123 | 0.413 | -0.926 | 0.510 | -0.998 | 0.824 | -1.000 | -0.884 |
|  |  | 500/1.0 | -0.510 | -0.441 | -0.441 | -0.986 | 0.779 | -0.989 | 0.490 | 0.277 | -0.875 | -0.657 |
|  |  | 650/0.5 | 0.247 | -0.470 | -0.257 | 0.955 | -0.940 | 0.986 | -0.861 | -0.924 | 0.282 | 0.996 |
|  |  | 650/1.0 | 0.114 | -0.185 | -1.000 | 0.967 | -0.604 | -0.924 | 0.110 | 0.920 | -0.981 | -0.614 |
|  |  | Control | 0.947 | 0.171 | 0.739 | -0.404 | 0.444 | 0.726 | 0.610 | 1.000 | 0.633 | 0.652 |
|  | 2-year after | 500/0.5 | 0.227 | -0.833 | -0.287 | 0.950 | -0.727 | 0.869 | -0.261 | -0.686 | 0.864 | -0.935 |
|  |  | 500/1.0 | 0.406 | -0.681 | 0.835 | -0.160 | 0.092 | -0.347 | 0.249 | -0.179 | 0.623 | 0.732 |
|  |  | 650/0.5 | 0.995 | 0.995 | -0.730 | 0.979 | 0.250 | -0.634 | 0.935 | -0.998 | 0.833 | -0.513 |
|  |  | 650/1.0 | 0.952 | 0.952 | -0.981 | -0.972 | -0.940 | 0.649 | 0.989 | -0.184 | 0.917 | 0.939 |
|  |  | Control | 0.859 | 0.859 | -0.942 | 0.326 | 0.137 | 0.708 | 0.705 | -0.912 | -0.334 | -0.125 |
